# The Constancy of Perceived Motion Under Different Spectral Conditions

**DOI:** 10.3390/vision9010015

**Published:** 2025-02-22

**Authors:** Jeffrey Nightingale, James M. Brown, Billy R. Hammond

**Affiliations:** 1 Vision Sciences Laboratory, Athens, GA 30602, USA; bhammond@uga.edu; 2 Visual Perception Laboratory, Athens, GA 30602, USA; jmbrown@uga.edu

**Keywords:** motion, constancy, motion constancy, light, vision, spectral filter, perception

## Abstract

(1) Background: Perceptual constancies are found in numerous categories of visual perception; color, lightness, and size constancy are notable examples where the perception of a visual scene remains constant, even with changing optical conditions. Constancies such as these are essential for survival, as they reduce the unpredictability of the world. In this study, we tested the resiliency of motion perception under widely differing spectral conditions. (2) Methods: Sixty healthy subjects (age range 18 to 26) were tested. Motion perception performance and thresholds were assessed using a novel, ecologically valid, psychophysical task implementing modern instruments. A broadband xenon bulb was used as a light source to emulate the spectral characteristics of natural daylight; 3 filter conditions were included to emulate different conditions of environmental light (short-wave, 400 nm–500 nm; medium-wave, 500 nm–600 nm; and long-wave, 600 nm–700 nm). (3) Results: In general, our findings showed that varying the spectral content of the broadband source did not change motion perception performance or thresholds for subjects. (4) Conclusions: These findings indicate that motion perception is highly resistant to changes in optical conditions, such as dramatically different spectral illuminants. This evidence is consistent with motion being considered among the perceptual constancies.

## 1. Introduction

Perceptual constancies are an integral feature of visual perception that allow humans to maintain a stable and accurate representation of the environment, despite changes in sensory input. Many categories of visual perception have constancies such as color, lightness, and size, to name a few. Using color constancy as an example, our visual system is able to perceive colors consistently, despite changes in illumination; similarly, size constancy is a feature of visual perception where an object’s perceived size remains constant at different distances. The main purpose of these constancies, as it relates to their function within the system of visual perception, is to reduce the unpredictability of the world. We live in a dynamic environment that is constantly changing, and perceptual constancies are what allow us to navigate the complex visual world with accuracy and consistency.

Green [1] provides a thorough overview of the challenges facing the mainstream interpretations and explanations of perceptual constancies, namely, that there are perceptual abilities that allow for stability of the visual scene through changes that do not fall under the classification of a perceptual constancy (ex. the ‘mere stability problem’). As noted by Green [1], there are three necessary components for perceptual constancy: perceptual variation, where sensory input changes due to alterations in viewing conditions; perceptual stability, where the perceived property of an object remains constant; and perceptual attribution, where stability is attributed to the object rather than external conditions.

In their paper on simultaneous color constancy, Arend and Reeves [2] demonstrate that neither spatial nor temporal factors alone are sufficient to maintain complete perceptual stability. Rather, a dynamic and coordinated interplay between these mechanisms is necessary to maintain consistency. Spatial mechanisms provide local adjustments (ex. spatial comparisons of chromatic signals from different parts of the visual field), while temporal adaptation offers a more global recalibration of perception in order to maintain consistency (ex. modification of chromatic channel sensitivity based on changing illuminants) [2]; spatial mechanisms work instantaneously to account for environmental variations, while temporal mechanisms build and recalibrate over time to provide stability across prolonged viewing.

Motion constancy [3] is characterized by the integration of motion properties (velocity, true object speed; speed, consistent rate of motion; displacement/position, coherent spatial movement) and is defined here as the ability to perceive consistent motion properties of objects, despite optical changes as a result of variations in visual conditions. Velocity, speed, and position are all terms that have been used in the visual perception and psychophysics literature to describe the broader concept of motion perception. While speed, velocity, and position constancy address isolated aspects of motion perception, motion constancy as a holistic framework emerges as a more comprehensive concept.

Velocity constancy can be defined as the ability to perceive the true speed of an object regardless of its distance from the observer. Wallach [4] was one of the first researchers to describe a constancy related to motion, and in doing so, introduced the transposition principle, arguing that the constancy of velocity arises when objects in different-sized frames are perceived as moving at the same speed—provided their retinal velocities are scaled proportionally. Much later, Zohary and Sittig [5] explored mechanisms of velocity constancy which emphasized a relative scaling algorithm, where retinal displacement is interpreted using scale references in the visual scene. Both of these studies provide a framework within which velocity constancy captures certain aspects of motion perception but is limited by its reliance on specific distance cues or spatial references, failing to address dynamic changes in the broader visual context.

Speed and position constancy similarly find themselves limited in accounting for the dynamic and contextual aspects of motion constancy, which captures the complex interactions between retinal velocity, object displacement, and perceived speed. Speed constancy can be defined as perceiving a consistent rate of motion of objects, despite variations in retinal velocity. McKee and Welch [6] and Tozawa [7] contrasted two hypotheses explaining speed constancy: the distance-calibration hypothesis, which attributes speed constancy to the integration of distance cues, and the relational hypothesis, which relies on the relative displacement of objects within a reference frame. Both frameworks offer some insights into the perception of speed, but do not capture the more complex interactions between motion properties. Position constancy can be defined as the ability to perceive an object as stationary or moving coherently within a spatial framework, despite shifts in the retinal image. Becklen, Wallach, and Nitzberg [8] described position constancy, and demonstrated that it is often compromised when target motion deviates from the direction of eye movement. Their experiments revealed that position constancy operates under specific conditions but fails when retinal and extraretinal cues conflict, again falling short of explaining constancy of the broader motion properties.

Past studies [3] have defined motion constancy in terms of perceived true linear speed but have failed to consider the integrative nature of motion properties such as velocity and displacement, in addition to being limited in the environmental validity of their methods in demonstrating the resiliency of motion under different conditions. In our study, we tested the consistency and resilience of motion perception under a variety of different optical conditions, using an ecologically valid design and psychophysical task.

We chose to vary spectral conditions based on past studies that have shown that many visual traits are influenced by varying wavelength (e.g., simple spectral sensitivity), especially in conditions of light stress. For example, Flannagan et al. [9] and Stringham et al. [10] showed that, all things equal, short-wave light is more aversive than mid-and-long wave light. We also chose to study the lower, vs. higher, threshold of motion. Past data on isochronal (slow) motion thresholds suggested that these thresholds may be particularly sensitive to optical factors [11].

Based on the literature findings [5,6,8] that motion properties (speed, velocity, displacement) demonstrate constancy under changing conditions such as retinal velocity [7] and distance [3], we hypothesized that motion perception performance and thresholds would remain consistent across varying spectral conditions. We tested this hypothesis using a modern adaptive psychophysical design and a variety of imposed lighting conditions (including those that one might encounter in real life, such as emulated daylight).

## 2. Materials and Methods

### 2.1. Subjects

Sixty healthy subjects participated (M age = 19.25, SD = 1.88 years; range = 8, 18 to 26). The sample consisted of 38 women and 22 men. Overall, 65% of the sample was white, 21.7% Hispanic, 21.7% Asian, and 6.7% Black. The target population for the present study consisted of healthy, young adults with normal or corrected-to-normal vision. Normal vision was set as 20/30 or better visual acuity in both eyes—first determined by self-report, and then verified using a wall-mounted Snellen chart. We found that 65% of the sample did not wear corrective lenses and 35% required correction to achieve 20/30 visual acuity. All study procedures and materials were approved by the University of Georgia Institutional Review Board prior to initiating the study. All participants gave both written and verbal informed consent prior to participation and the tenets of the Declaration of Helsinki for research on human subjects were followed.

### 2.2. Design

**Figure 1:**
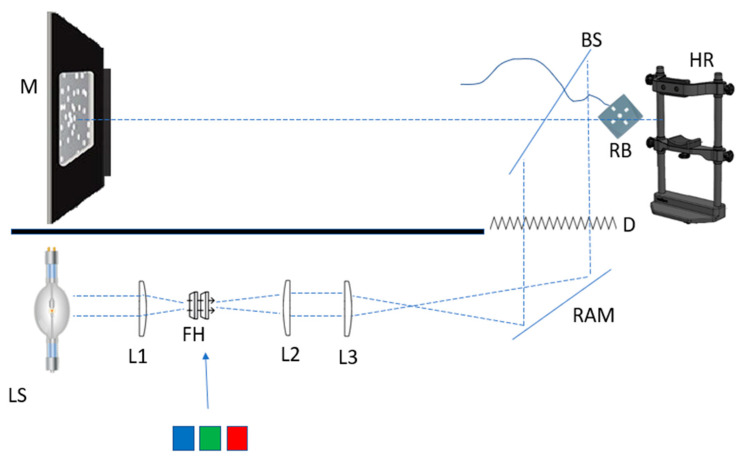
LS, light source, M, monitor, BS, beam splitter, RB, response box, HR, head rest, D, diffuser, RAM, right angle mirror, FH, filter holder.

Motion perception thresholds were evaluated using a forced-choice direction discrimination task conducted through a hybrid system combining the Metropsis (SKU M0400, version 2.5.1) laboratory setup and an optical bench. Figure 1 provides a conceptual representation of the apparatus. The experiment was divided into three parts, each varying in viewing conditions:

- Part 1: Participants performed the motion perception task without any veiling light.
- Part 2: A Xenon white, unfiltered light emulating daylight conditions was introduced.
- Part 3: Participants completed the task under three spectrally filtered lighting conditions: short-wave, medium-wave, and long-wave veiling light.

The Metropsis system (SKU M0400, version 2.5.1), operated via an iMac desktop computer and monitored remotely using an iPad, was used to control the experiment and track participants’ responses. The primary task involved a random dot kinematogram (RDK) created using Psykinematix software (GPU Edition, version 2.7). In this task:

- 100 white dots, with maximum luminance of 350 cd/m² and 100% coherence, were displayed against a black background of zero luminance.
- The dots moved randomly in one of four directions (up, down, left, or right) at a starting speed ranging between 0.01 and 5 degrees/second for a duration of one second.
- Each dot’s visibility decayed asynchronously over the presentation period.
- Dot density was 100 dots on a 21 deg area, viewed at a distance of 1.5 m.

Participants were seated in a completely darkened room, approximately 1.5 m away from the stimulus display, with their head stabilized in a chinrest to ensure consistent binocular viewing. A clear glass pane was placed in their line of sight. For Part 1, the glass did not affect visibility. For Parts 2 and 3, the glass acted as a beam-splitter, introducing light from a veiling source into the participant’s view.

The participants’ task was to identify the direction of motion of the dots in each trial by pressing one of four buttons on a Cedrus response box. The button pressed corresponded to the perceived direction (ex. top button for upward motion, bottom button for downward motion, and side buttons for left or right motion). The experiment employed a staircase method, requiring participants to complete multiple trials until their motion perception threshold was determined. This methodology allowed for precise measurement of their ability to discriminate motion direction under various lighting conditions.

### 2.3. Instruments

The optical bench setup and direction discrimination task combined established methodologies in motion perception research [12,13,14,15,16] with an ancillary optical system.

**Figure 2:**
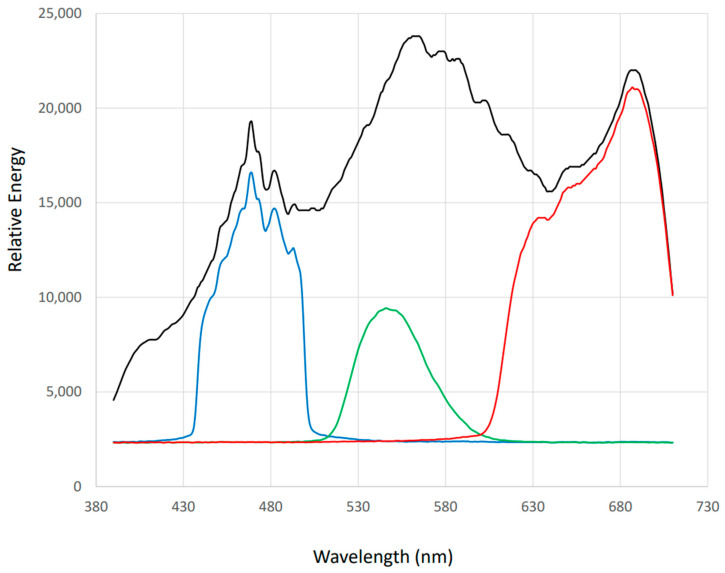
Xenon white (black), short-wave (blue), medium-wave (green), and long-wave (red) spectral graphs.

To assess the spectral properties of the light source and the wavebands for each filter condition, an ILT960-BB Spectroradiometer was employed. Figure 2 displays the spectral graphs for the light source, including the Xenon white, short-wave, medium-wave, and long-wave filter measurements. An LED light meter was utilized to confirm that light levels remained consistent across all conditions. The light meter was positioned at eye level during measurements, ensuring that the recorded energy levels were uniform across conditions (M = 87 lux).

### 2.4. Procedure

Prior to initiating an experimental session, several preparatory steps were completed to ensure accurate and consistent results. These included verifying the correct alignment of all optical components, confirming the appropriate light intensity of the Xenon source, and calibrating the stimulus display using the Display++ real-time calibration procedures (with no error reports). Additionally, the participant’s head was securely positioned in the chinrest to maintain stability throughout the session.

The direction discrimination task was then thoroughly explained to the participant before presenting the stimuli. The primary objective of the experimental design was to measure motion perception performance, quantified as the percentage of correct responses out of the total trials completed, and to determine LTM (lower threshold of motion) speed thresholds, defined as the lowest speed (in degrees/second) at which a correct response was recorded. These measurements were obtained across all three parts and for each of the lighting conditions in Part 3.

Motion perception was measured, using a staircase method to determine motion perception performance and LTM speed thresholds for the direction discrimination task. The RDK’s direction was randomized, and its speed was adjusted incrementally to obtain performance and threshold values. Participants were seated at one end of an optical table and positioned in a chinrest to maintain head stability. The stimulus display (Display++) was fixed approximately 1.5 m away at the opposite end of the table. Before beginning the task, the researcher explained the direction discrimination procedure to the participant. A random number generator determined the starting part (1, 2, or 3) and the order of spectral filter conditions (short-wave, medium-wave, long-wave).

## 3. Results

### 3.1. Descriptive Statistics

Table 1 presents the descriptive statistics for all demographic variables in the study. The sample consisted of a relatively homogenous group of young adults (M age = 19 years, SD = 1.88).

### 3.2. Resilience of Motion

A one-way, within-subjects ANOVA was conducted to examine the effect of spectral filtering on motion perception performance across the no veil, white veil, short-wave, medium-wave, and long-wave conditions. The analysis revealed no significant effect of the separate lighting conditions on performance scores, *F*(4, 236) = 0.87, *p* = 0.48. The means and standard deviations for each filter condition were as follows: long-wave (M = 78.64, SD = 7.57), medium-wave (M = 77.02, SD = 8.29), no glare (M = 77.43, SD = 7.02), short-wave (M = 77.99, SD = 6.91), unfiltered (M = 78.72, SD = 7.25).

Similarly, another one-way, within-subjects ANOVA was used to evaluate the influence of spectral filtering on LTM speed thresholds across the same conditions. The results indicated no significant effect of the lighting conditions on LTM speed thresholds, *F*(4, 236) = 0.63, *p* = 0.61. The means and standard deviations for each filter condition were as follows: long-wave (M = 0.16, SD = 0.37), medium-wave (M = 0.11, SD = 0.11), no glare (M = 0.21, SD = 0.18), short-wave (M = 0.24, SD = 1.08), unfiltered (M = 0.21, SD = 0.69).

**Figure 3:**
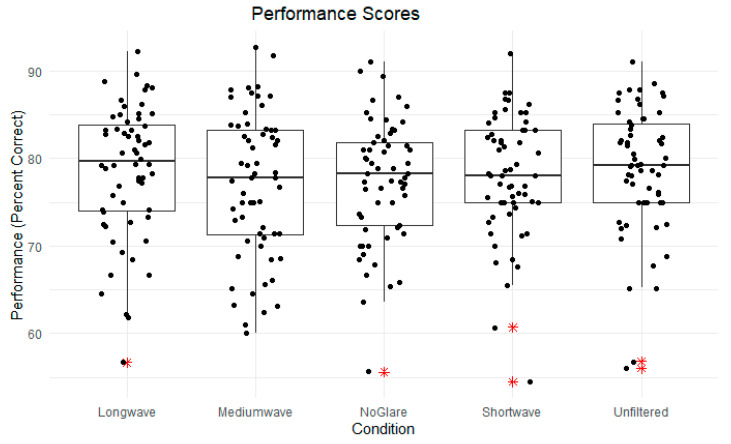
Box plots of performance scores, organized by filter condition. Center line denotes median performance score. * Means are all non-significant.

**Figure 4:**
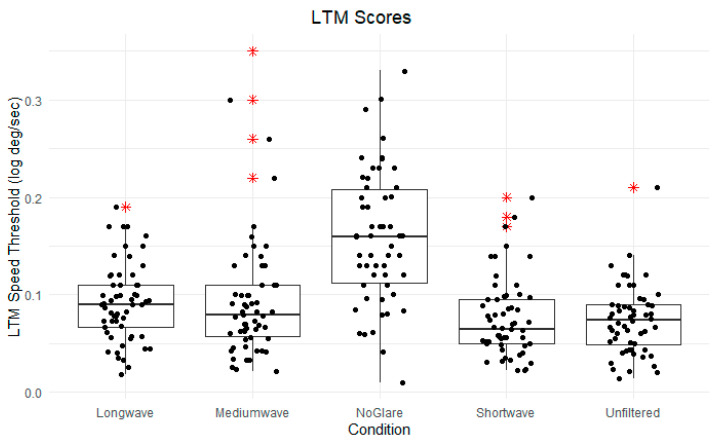
Box plots of LTM scores, organized by filter condition. Center line denotes median LTM score. * Means are all non-significant.

Box plots depicting the mean scores for performance and LTM thresholds by filter condition are presented in Figure 3 and Figure 4.

Pearson correlations were conducted for all combinations of filter pairs to look at individual covariation across conditions. The results of these correlations are shown in Table 2 (performance scores) and Table 3 (LTM speed thresholds). Nearly all filter pairs show a significant correlation.

A series of paired-sample t-tests were performed to investigate pairwise differences between the no glare and filtered conditions for both performance scores and LTM thresholds. No significant differences in performance scores were found between the no glare condition and any of the filter conditions: no glare/unfiltered (*t* = −1.24, *p* = 0.22), no glare/short-wave (*t* = −0.55, *p* = 0.58), no glare/medium-wave (*t* = 0.35, *p* = 0.73), no glare/long-wave (*t* = −1.0, *p* = 0.32).

However, results indicated that for LTM, speed thresholds scores in the no glare condition (M = 0.21, SD = 0.18) were significantly higher than those in the medium-wave condition (M = 0.11, SD = 0.11), *t*(59) = 4.94, *p* < 0.001. No other significant differences were found in LTM scores between the no glare and filtered conditions: no glare/unfiltered (*t* = 0.01, *p* = 0.99), no glare/short-wave (*t* = −0.22, *p* = 0.82), no glare/long-wave (*t* = 1.2, *p* = 0.23).

A summary of the paired-sample t-tests is presented in Table 4 for performance scores and Table 5 for LTM speed thresholds.

## 4. Discussion

In the present study, we examined motion perception performance and thresholds under varying visual conditions. Our findings revealed that motion perception, for our conditions, remains stable across a diverse range of emulated environmental lighting conditions. This consistency was robust. Despite the stimuli being viewed in the absence of light stress, under very bright white light conditions (emulated glaring daylight) or broad-band blue, green, or red, the average thresholds were highly consistent.

The absence of significant differences in motion perception performance and thresholds across the no veil, white veil (i.e., glare), and spectral conditions demonstrates the robustness of the mechanisms that contribute to motion constancy. This stability aligns with the criteria outlined by Green [1] for perceptual constancies: perceptual variation, perceptual stability, and perceptual attribution. The observed resilience of motion perception across diverse lighting conditions fulfills these criteria, suggesting that motion constancy serves as an adaptive mechanism to maintain consistent motion perception in a dynamic environment with ever-changing visual conditions.

It is possible that variations in the upper threshold of motion could be present across the varying lighting conditions used in this study. Future studies should aim to investigate the upper threshold of motion to see if the consistency seen in the lower threshold of motion across lighting conditions is maintained. Such investigations could include the potential effects of different stimulus parameters (ex. dot density, dot background contrast, motion duration) on motion perception performance and LTM speed thresholds.

While previous studies have provided foundational insights into the constancy of individual motion properties [4,6], our findings offer a more integrative perspective by demonstrating that motion constancy encompasses the broader interplay of velocity, speed, and displacement. This holistic view supports the notion that motion constancy extends beyond isolated attributes to include the complex interactions of motion properties in real-world scenarios. By employing a hybrid optical bench and random dot kinematogram (RDK) paradigm, we were able to simulate realistic visual conditions while maintaining experimental control. This study demonstrates that—just like color, lightness, and size—motion perception is remarkably stable across varying optical conditions, providing strong evidence for motion constancy as an adaptive feature of visual perception.

## Figures and Tables

**Table 1:** Descriptive statistics (*n* = 60).

Variable	Average	SD	Range	n Analyzable
Age	19.25	1.88	8, 18 to 26	60
MPOD (10′)	0.48	0.19	1, 0 to 1	60
Race	Asian—21.7%	N/A	N/A	60
	Black—6.7%			
	Hispanic—21.7%			
	White—65.0%			
Sex	Female—63.3%	N/A	N/A	60
	Male—36.7%			
Vision Correction	Correction—35%	N/A	N/A	60
	No Correction—65%			
Iris Color (Hue)	Blue—28.3%	N/A	N/A	60
	Brown—43.3%			
	Gray—3.3%			
	Green—15%			
	Hazel—10%			
Iris Color (Lightness)	Dark—33.3%	N/A	N/A	60
	Light—28.3%			
	Medium—38.3%			

**Table 2:** Pearson correlations pairs (performance).

Variable	Df	Correlation	Significance
No Glare/Unfiltered	58	0.36	0.005 **
No Glare/Short-wave	58	0.36	0.004 **
No Glare/Medium-wave	58	0.31	0.01 **
No Glare/Long-wave	58	0.18	0.16
Unfiltered/Short-wave	58	0.49	<0.001 ***
Unfiltered/Medium-wave	58	0.27	0.04 *
Unfiltered/Long-wave	58	0.37	0.003 **
Short-wave/Medium-wave	58	0.17	0.20
Short-wave/Long-wave	58	0.38	0.003 **
Medium-wave/Long-wave	58	0.26	0.05 *

**Table 3:** Pearson correlation pairs (LTM speed thresholds).

Variable	Df	Correlation	Significance
No Glare/White Veil	58	0.65	<0.001 ***
No Glare/Short-wave	58	0.59	<0.001 ***
No Glare/Medium-wave	58	0.46	<0.001 ***
No Glare/Long-wave	58	0.56	<0.001 ***
Unfiltered/Short-wave	58	0.97	<0.001 ***
Unfiltered/Medium-wave	58	0.42	<0.001 ***
Unfiltered/Long-wave	58	0.45	<0.001 ***
Short-wave/Medium-wave	58	0.32	0.01 **
Short-wave/Long-wave	58	0.34	0.008 **
Medium-wave/Long-wave	58	0.72	<0.001 ***

**Table 4:** Paired-sample t-tests (performance).

Filter Pair	Df	t	Significance
No Glare/Unfiltered	59	−1.24	0.22
No Glare/Short-wave	59	−0.55	0.58
No Glare/Medium-wave	59	0.35	0.73
No Glare/Long-wave	59	−1.0	0.32

**Table 5:** Paired-sample t-tests (LTM speed thresholds).

Filter Pair	Df	t	Significance
No Glare/Unfiltered	59	0.01	0.99
No Glare/Short-wave	59	−0.22	0.82
No Glare/Medium-wave	59	4.94	<0.001 ***
No Glare/Long-wave	59	1.2	0.23

## Data Availability

The original contributions presented in this study are included in the article. Further inquiries can be directed to the corresponding author.

## Abbreviations

The following abbreviations are used in this manuscript:

RDK	Random Dot Kinematogram
LTM	Lower Threshold of Motion
